# Yields and resilience outcomes of organic, cover crop, and conventional practices in a Mediterranean climate

**DOI:** 10.1038/s41598-019-48747-4

**Published:** 2019-08-22

**Authors:** Meng Li, Caitlin A. Peterson, Nicole E. Tautges, Kate M. Scow, Amélie C. M. Gaudin

**Affiliations:** 1 Department of Plant Sciences, University of California, Davis, One Shields Avenue, Davis, CA 95616 United States; 2 Agricultural Sustainability Institute, University of California, Davis, One Shields Avenue, Davis, CA 95616 United States; 3 Department of Land, Air, and Water Resources, University of California, Davis, One Shields Avenue, Davis, CA 95616 United States

**Keywords:** Agroecology, Ecosystem services

## Abstract

Adaptive management practices that maximize yields while improving yield resilience are required in the face of resource variability and climate change. Ecological intensification such as organic farming and cover cropping are lauded in some studies for fostering yield resilience, but subject to criticism in others for their low productivity. We implemented a quantitative framework to assess yield resilience, emphasizing four aspects of yield dynamics: yield, yield stability, yield resistance (i.e., the ability of systems to avoid crop failure under stressful growing conditions), and maximum yield potential. We compared the resilience of maize-tomato rotation systems after 24 years of irrigated organic, cover cropped, and conventional management in a Mediterranean climate, and identified crop-specific resilience responses of tomato and maize to three management systems. Organic management maintained tomato yields comparable to those under conventional management, while increasing yield stability and resistance. However, organic and cover cropped system resulted in 36.1% and 35.8% lower maize yields and reduced yield stability and resistance than the conventional system. Our analyses suggest that investments in ecological intensification approaches could potentially contribute to long-term yield resilience, however, these approaches need to be tailored for individual crops and systems to maximize their benefits, rather than employing one-size-fits-all approaches.

## Introduction

Growing consensus suggests that approaches to food production centered only on maximizing yield hampers the future sustainability of our agroecosystems by not optimizing whole-ecosystem performance^1^. Large environmental externalities coupled with shifts in resource availability associated with climate change require the adoption of adaptive management practices that improve productivity but also yield resilience, while maintaining environmental integrity. Managing for resilience is often equated with reducing reliance on external inputs by harnessing self-regulation from ecosystem processes, and providing reliable ecosystem services under abiotic and biotic stresses^2,3^. To be of value in intensively managed agroecosystems, managing for resilience should maintain high yields and increasing trends while also addressing yield stability (i.e., low year-to-year variability) and yield resistance (i.e., low potential of crop failure) in the face of adverse biotic and abiotic shocks^4^. However, production systems and their analyses have historically focused on absolute yields, and yield stability and resistance dimensions are seldom considered.

Ecological intensification practices are often cited as strategies to nudge production systems towards resilient states by leveraging ecosystem processes rather than external chemical and energy inputs^5^. Diversification strategies such as cover crops and whole-system approach, like organic management, both fall under the umbrella of ecological intensification because they have the potential to reduce or replace the use of synthetic inputs with ecosystem services^6^. Cover cropping, especially when it include a leguminous species, have the potential to reduce nitrogen inputs while improving soil quality, conserving soil moisture, and suppressing weeds in a variety of systems^7,8^. Organic farming approaches have also been shown to support above- and below-ground biodiversity, enhance soil quality, regulate soil nutrient cycling processes, and improve climate change mitigation potential of cropping systems, especially in California, which leads the organic production in the United States^9–11^. Despite these ecological benefits of relevance to build up stress resilience in multiple contexts^12^, adoption rates of organic and cover cropping practices remain low due to concerns over lower yields compared to more conventional production approaches^13–15^. However, short-term productivity gains can wane over time without favorable climates and policies or continued access to affordable and plentiful external inputs^16^. The potential of agricultural practices to build up resilience to multiple stressors and to provide more stable yields is becoming increasingly important.

So far, studies comparing conventional and ecological intensification systems have mainly focused on their differences in ecosystem services and absolute yields, and only a few have reported yield resilience metrics of these comparisons^10,17,18^. Some studies have shown reduced yield stability in organic or cover cropped systems in cases of biotic stresses from weed, pathogen, or insect pest outbreaks^19,20^. Depending on systems features, other studies provide evidence that ecological intensification systems achieve biological control of some pests via increased above- and belowground biodiversity^21^. Both organic and cover cropping have also been reported to build systemic buffering capacity and increase yield resistance to extreme weather conditions, especially drought, thanks to the accumulation of soil organic carbon (SOC) and resultant increases in water holding capacity in rainfed systems^22,23^. These contrasting reports of the relative benefits of conventional vs. ecological intensification systems highlight the need for more analyses of long-term yield dynamics and resilience, especially for highly vulnerable irrigated systems in semi-arid landscapes, which remain understudied. In Mediterranean climates such as California, where extended droughts are common and irrigated systems make up the majority of agricultural production by value, improved resource use efficiency and resilience are critical for the overall sustainability of agricultural production in a changing climate.

We analyzed yield data collected in the first 24 years of a long-term irrigated farming systems experiment located in a semi-arid Mediterranean climate. Borrowing concepts from ecosystem resilience adapted for production based agroecosystems^4^, we propose a novel framework to compare the impacts of management on long-term yield resilience. It is composed of four core aspects: (1) absolute yields and yield trends, (2) yield stability, (3) yield resistance, and (4) maximum yield potentials. Analysis of absolute yields and their trends over time can reveal a warning signal for changes in system state resulting from climate change and management-induced changes in ecosystem processes that might impair or improve resilience in the long term^24^. Yield stability measures the ability of agricultural systems to maintain consistent yields over time despite normal environmental variation, such as temperature and rainfall fluctuations^25,26^. Yield resistance reflects the ability of agricultural systems to mitigate the risk of crop failure due to unexpected abiotic and biotic shocks, while maximum yield potential measures the ability of systems to produce high yields when conditions are optimum^26,27^. Both yield resistance and maximum yield potential can be measured by quantifying the probability of the yield falling into a certain threshold or the potential of yields to decline and increase under adverse and optimum environmental conditions^26,27^. Analyzed together, these metrics and properties could serve as a launchpad for further research into the practices and mechanisms involved in long-term yield resilience.

We applied these concepts to the analysis of irrigated maize-tomato long-term rotations under three different management systems: a conventional management system, and two ecological intensification strategies. We hypothesized that thanks to enhanced soil and ecosystem services, long-term ecological intensification strategies such as organic management and cover crops will: (1) increase crop yields over time and perform comparably to conventional systems in term of absolute crop yields; and (2) increase system resilience as measured by enhanced yield stability and resistance under adverse environmental conditions. This novel approach will provide a strong foundation for future analysis of resilience across cropping systems and provide insights into the design of sustainable cropping systems that go beyond yield maximization to consider resilience.

## Materials and Methods

### Site description

The Century Experiment located at the Russell Ranch Sustainable Agricultural Facility (RRSAF) has been ongoing since 1993 and is set to run for 100 years. It is composed of 11 farming systems replicated over 72, 0.4 ha plots managed by the University of California, Davis and located in Winters, California (38.54′N, 121.87′W). Prior to cultivation, the site was a grassland with average initial soil organic matter (SOM) of 1.23%. In 1992 and 1993, sudangrass (*Sorghum bicolor* (L.) Moench) was planted to increase soil uniformity prior to trial establishment^28^. The climate is Mediterranean, characterized by mild and rainy winters and hot dry summers. Annual average atmospheric temperature and precipitation (1994–2017) are shown in Supplementary Fig. S1. The experimental plot used for this study spans two soil types: Rincon silty clay loam (fine, montmorillonitic, thermic Mollic Haploxeralfs) to the north and Yolo silt loam (fine-silty, mixed nonacid, thermic Typic Xerorthents) to the south^29^.

### Experimental design and system management

Maize (*Zea mays* L.) and processing tomato (*Solanum lycopersicum* L.) rotations were implemented in 1994 with both rotation entry points present each year. The experiment is a completely randomized block design with three management systems applied to the maize/tomato rotation: (1) conventional maize-tomato system with synthetic inputs (“CONV”), (2) maize-tomato system with synthetic inputs and winter cover crops (“CONV + WCC”), and (3) organic maize-tomato system with annual compost applications, winter cover crops, and no synthetic inputs (“ORG”). Each system has six replicates: three replicates for maize and three for tomato within each year.

The CONV system received synthetic fertilizers annually, on average 200 kg N ha^−1^ and 235 kg N ha^−1^ for tomato and maize, respectively (Supplementary Table S1). For the CONV + WCC system, synthetic fertilizers were applied only to the tomato phase from 1994 to 2008, and both tomato and maize were fertilized starting in 2009. WCC mixes were planted in the fall only before maize (1994–2004), and before both crops thereafter (2005–2017) in this system. Thus, fertility input levels in CONV + WCC system were not a mirror of the CONV system, especially in the early years of this experiment. The ORG system received chicken manure compost annually, composed of approximately 20.0% carbon (C), 2.0% nitrogen (N), and 1.4% phosphorus (P) prior to planting. WCC mixes were planted every fall prior to tomato and maize crops in ORG. The WCC planted in the CONV + WCC and ORG systems was a mix of 90 kg ha^−1^ of Magnus peas (*Pisum sativum* L.) and 45 kg ha^−1^ of lana vetch (*Vicia villosa* Roth) from 1994 to 2005. From 2006 to 2017, the WCC mixes were diversified to 90 kg ha^−1^ bell bean (*Vicia faba* L.), 22.5 kg ha^−1^ lana vetch, and 28 kg ha^−1^ oats (*Avena sativa* L.). All three systems received furrow-flood irrigation from 1994 to 2014. Starting in 2015, the CONV and CONV + WCC plots were converted to subsurface drip irrigation, and ORG plots were divided into two irrigation treatments (furrow and subsurface drip irrigation) for long-term comparison. The yields presented here for the ORG system are the results from furrow-irrigated subplots. More detailed management practices for the three systems are given in Supplementary Table S1 and also accessible in the published dataset^29^.

Four randomly selected tomato rows from each plot were mechanically harvested each year, and yields were calculated based on the fresh weight of marketable fruits per unit area. For maize, yields were determined as the moisture adjusted dry weight of grain from mechanically harvested rows. The CONV + WCC system was fallowed in 2008 (no tomato yields available), and maize yields are unavailable from 2008–2012 for all three systems as maize was replaced by sudangrass in 2009 and wheat (*Triticum aestivum* L.) from 2010–2012^29^.

### Data analysis

#### Mean yields and trends

All statistical analyses were performed in R (3.4.1)^30^. The function ‘cld’ in the R package ‘lsmeans’ was used to conduct multiple mean comparisons^31^. The functions ‘ACF’ and ‘lme’ in the R package ‘nlme’ were used to check for autocorrelation of residual structures and to conduct linear mixed-effect models respectively^32^.

Long term mean maize and tomato yields of the three systems were analyzed using a linear mixed-effects model with cropping system as the fixed effect, and year and block as random effects. Post-hoc Tukey multiple comparisons of means were applied to compare mean yields across the three systems with confidence intervals adjusted using the Sidak method.

Maize and tomato yield trends over 24 years were analyzed using linear mixed-effects models with cropping system and year as fixed effects and block as a random effect. Likelihood ratio tests were conducted to choose the most parsimonious models based on tests of different random structures and temporal autocorrelation of residuals. For both tomato and maize, the most parsimonious model contained no temporal autocorrelation residual structures. The three management systems were allowed to have different within-group variances in the model to account for non-homogeneity of variance. The assumption of normal distribution of residues was verified with the Shapiro-Wilk normality test. Results of the analysis of variance for the chosen models are reported in Supplementary Table S2. For maize, yield patterns were analyzed separately for the periods 1994–2007 and 2012–2017 due to the missing data.

#### Yield stability

Four yield stability metrics per system were calculated and compared for both maize and tomatoes: (1) yield range, (2) coefficient of variation (CV), (3) yield variance, and (4) Finlay-Wilkinson (FW) regression slope^33^. Yield range represents the range between the highest and lowest yield of each system across the dataset. The other three stability metrics were obtained based on the de-trended yield data (i.e., residuals from regressing yields against year with system-specific intercepts and slopes) to remove potential biases from yield increases associated with genetic and technological improvements over time. The CV was calculated by dividing the temporal standard deviation by the mean yield. Yield variance represents the temporal variance over 24 years. FW regression slopes were obtained by regressing de-trended yields of each system to the environmental index (EI). EI is expressed as the average of annual de-trended yields of tomato or maize over three systems^33^ and is used as an indication for the overall yielding ability at the respective environmental condition. Systems with smaller yield range, CV, yield variance, and FW slopes indicate higher yield stability. The overall yield stability of each cropping system was ranked based on the mean stability rank for the four stability metrics.

#### Yield resistance

Yield resistance is a key property of resilience in intensive systems and represents the ability of systems to avoid crop failure under stressful growing conditions^4,34^. Tomato yield resistance was calculated using two metrics: (1) probability of crop failure based on frequency distributions^26^, and (2) predictions of minimum yields according to EIs^27^.

Probabilities of low yields were performed by estimating the probability densities of tomato yields in each cropping system and the pooled dataset using kernel distribution. The probabilities of three systems to achieve low yields (<10^th^ percentile of the pooled yield distribution estimate) were extracted. The significance of the probabilities of low yields was determined by comparing each system to the probabilities of low yields from 5,000 randomized yield sets. The pseudo-p for low yield probability represents the percent times that each system would yield lower than the distribution of randomized yields using a left-tail test. The probability of low maize yield was not analyzed due to the missing data and small sample size (n = 19). The second method compared the predictions of minimum yields of tomato and maize in three systems under unfavorable growing conditions (lowest EI) based on a linear mixed-effects model with EI and system as fixed effects and block as the random effect. To indicate actual yield ranges, de-trended yields were re-centered to the mean yields of each system (i.e., adding system mean yields to de-trended data).

#### Maximum yield potential

Using the same method as yield resistance, we also estimated the probabilities of tomato to obtain high yields (>90^th^ percentile of the pooled yield distribution estimate) in the three systems, and the maximum yield potential of tomato and maize under favorable conditions (highest EI). This measurement can help indicate management-induced differences in the potential of tomato and maize to capitalize on favorable growing conditions.

## Results

### Mean yields and trends

**Figure 1:**
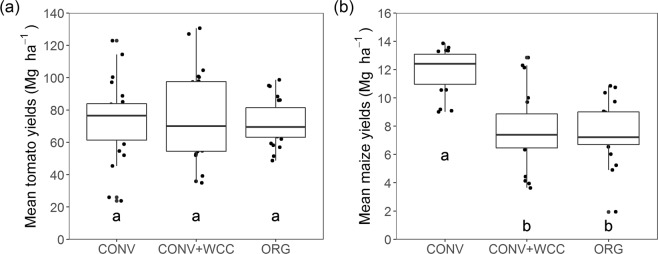
Mean yields of tomato (**a**) and maize (**b**) in the maize-tomato rotation system from 1994 to 2017. CONV: conventional maize-tomato system; CONV + WCC: maize-tomato system with winter cover crops; ORG: organic maize-tomato system. Letters represent significant differences in mean yields at the 0.05 significance level.

**Figure 2:**
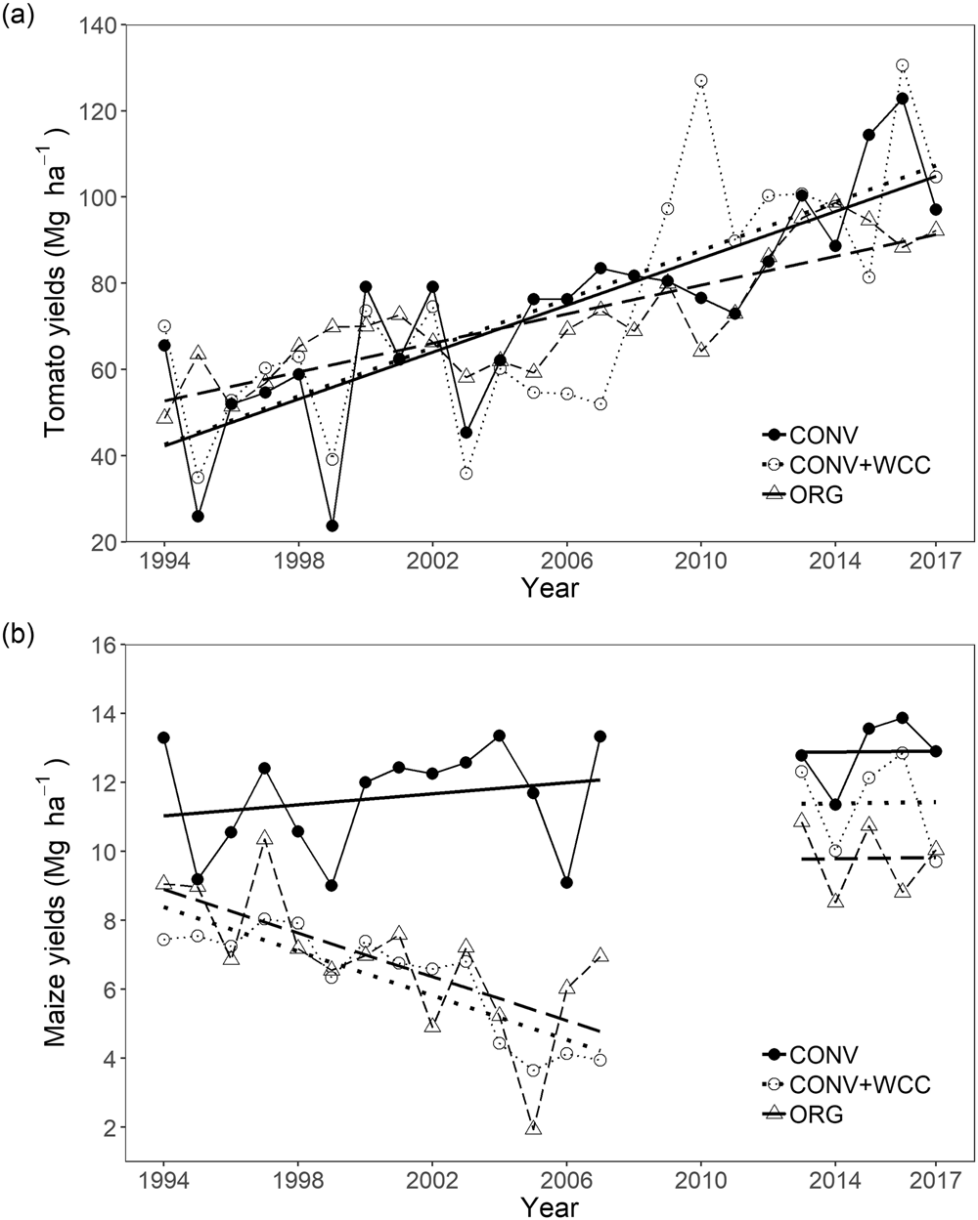
Interaction plot showing long-term yield trajectories of tomato (**a**) and maize (**b**) in the maize-tomato rotation system from 1994 to 2017. CONV: conventional maize-tomato system; CONV + WCC: maize-tomato system with winter cover crops; ORG: organic maize-tomato system. Solid circles and solid lines = CONV; open circles and dotted lines = CONV + WCC; open triangles and long-dashed lines = ORG.

Mean tomato yields (1994–2017) were not significantly different between the three systems (F_2, 208_ = 0.578, p = 0.562; Fig. 1a). However, systems responded differently to yearly variation as indicated by a significant system by year interaction term (Fig. 2a; Supplementary Table S2). The rate of yield increases for tomato differed across the three systems and was slower in ORG (1.680 Mg ha^−1^ per year) than in CONV (2.721 Mg ha^−1^ per year, p < 0.001) and CONV + WCC (2.815 Mg ha^−1^ per year, p = 0.004). Yield trends were similar between CONV and CONV + WCC (p = 0.835; Fig. 2a).

Conversely, management system had a significant impact on mean maize yields (F_2, 166_ = 105.652, p < 0.001), with ORG and CONV + WCC attaining maize yields 36.1% and 35.8% lower than the CONV, respectively (Fig. 1b). From 1994 to 2007, trends of maize yield differed significantly between the three systems, as indicated by a significant system by year interaction term (Fig. 2b; Supplementary Table S2). Maize yields in ORG and CONV + WCC declined sharply during this period (ORG: slope = −0.317, p =  < 0.001; CONV + WCC: slope = −0.321, p = <0.001), while there was no significant trend in yields of CONV (slope = 0.081, p = 0.192; Fig. 2b). From 2012 to 2017, maize yields significantly responded to system management (Supplementary Table S2), with CONV mean yields (12.889 Mg ha^−1^) on average 31.6% and 13.1% higher than the ORG (9.795 Mg ha^−1^) and CONV + WCC (11.398 Mg ha^−1^) respectively. There was no significant interaction effect between year and system for this period of time (Supplementary Table S2).

### Yield stability

**Figure 3:**
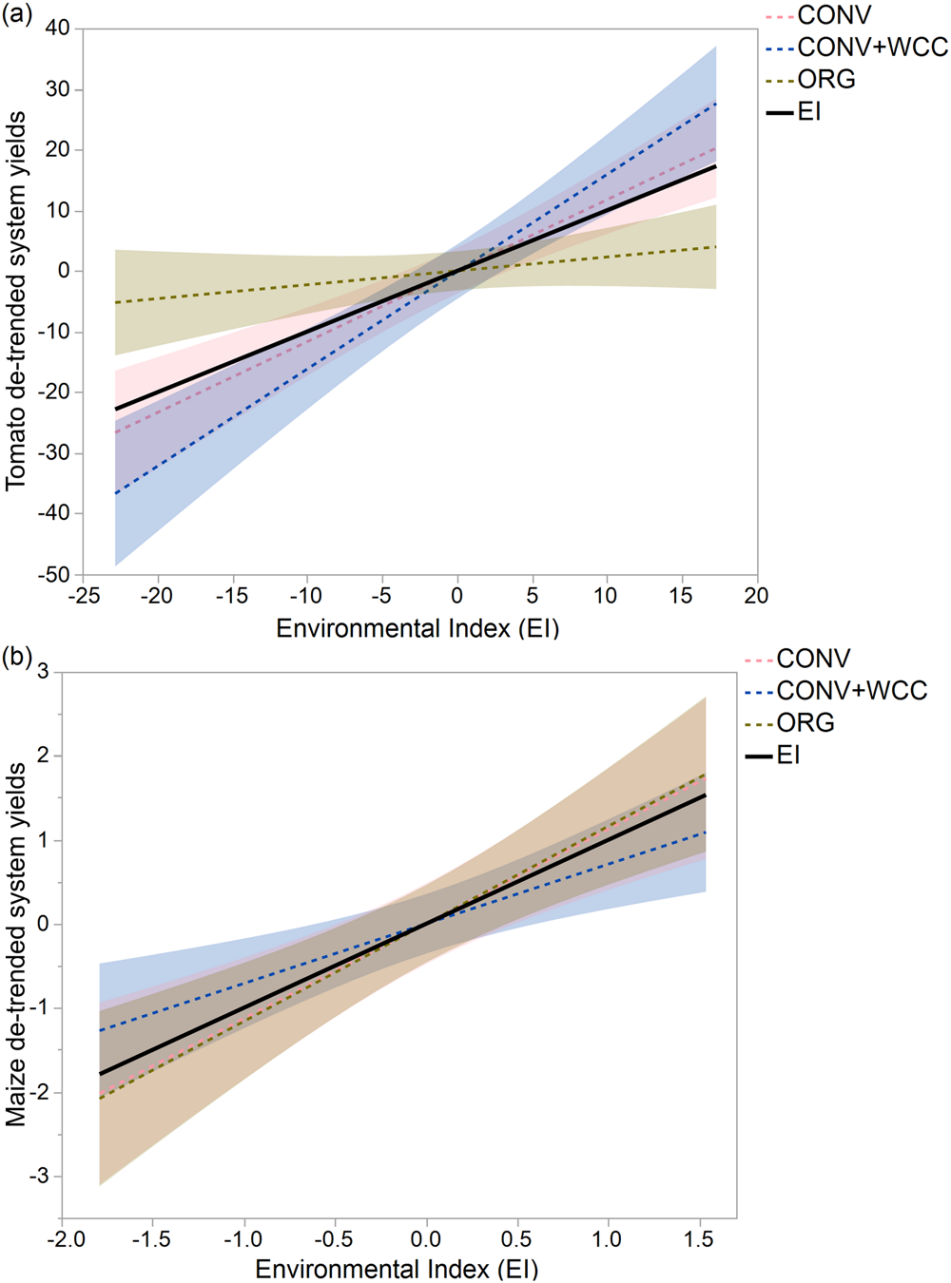
Yield stability of tomato (**a**) and maize (**b**) by regressing system-level de-trended yields of each system against environmental index (EI) calculated as the yearly mean de-trended yield. CONV: conventional maize-tomato system; CONV + WCC: maize-tomato system with winter cover crops; ORG: organic maize-tomato system. Smaller slopes indicate higher yield stability.

Tomato yield was the most stable in the ORG system with the lowest yield range, CV, yield variance, and FW slopes of the three systems (Table 1). CONV had the largest yield range, but ranked second in terms of CV, yield variation, and FW regression slope (Table 1; Fig. 3a). CONV + WCC was the least stable system with the largest FW slope of three systems, a one-fold higher CV (F_2, 5_ = 10.231, p = 0.017) and 108.7% higher yield variance than the ORG (F_2, 5_ = 10.780, p = 0.015), and 39.6% and 42.9% higher CV and yield variance than the CONV. Averaging over ranks of four stability metrics, tomato yield was the most stable in ORG following by CONV and was the least stable in the CONV + WCC system (Table 1).

**Table 1:** Yield stability parameters and ranks for tomato and maize yields in the maize-tomato rotation in three systems from 1994 to 2017.

Crop	System	Yield stability parameters				Rank
		Yield range	CV (%)	Yield variance	FW slope	
Tomato	ORG	50.044 (1)	0.142 a (1)	10.151 a (1)	0.216 (1)	1
	CONV	99.071 (3)	0.202 ab (2)	14.827 ab (2)	1.171 (2)	2.3
	CONV + WCC	95.680 (2)	0.284 b (3)	21.190 b (3)	1.616 (3)	2.8
Maize	CONV	4.858 (1)	0.123 a (1)	1.468 b (2)	1.131 (2)	1.5
	CONV + WCC	9.204 (3)	0.149 a (2)	1.133 a (1)	0.706 (1)	1.8
	ORG	8.919 (2)	0.206 b (3)	1.561 b (3)	1.163 (3)	2.8

CONV: conventional maize-tomato system; CONV + WCC: maize-tomato system with winter cover crops; ORG: organic maize-tomato system. Numbers in parentheses represent ranks of individual yield stability metrics for three systems. Letters represent significant differences among systems at the 0.05 significance level. CV represents coefficient of variation, and FW slope represents the Finlay and Wilkinson regression slope.

Conversely, maize yields were the most stable in the CONV system and least stable in the ORG system across years (Table 1). ORG system had significantly higher CV than both CONV and CONV + WCC (F_2, 5_ = 33.302, p = 0.001), but comparable yield variance to CONV. Yield variance in CONV + WCC was significantly lower than in CONV and ORG (F_2, 5_ = 18.246, p = 0.005). ORG and CONV had similar FW slopes, and CONV + WCC system was the lowest (Fig. 3b). Overall, maize yield stability was slightly higher in CONV than CONV + WCC, with the ORG system ranking last.

### Yield resistance

For tomato, ORG management significantly decreased the risk of low yields with the lowest probability of crop failure (<10^th^ percentile): 3.8% (pseudo-p = 0.024) compared to 12.4% for the CONV system and 19.2% for CONV + WCC (Table 2). Similarly, ORG was more resistant to unfavorable conditions than the other two systems as indicated by a higher tomato yield (67.201 Mg ha^−1^) than CONV (49.082 Mg ha^−1^) and CONV + WCC (40.581 Mg ha^−1^) under the lowest EI (Table 2). For maize, on the contrary, ORG (5.693 Mg ha^−1^) and CONV + WCC (6.483 Mg ha^−1^) were less resistant to unfavorable conditions than CONV (10.045 Mg ha^−1^) under the lowest EI (Table 2).

**Table 2:** The probabilities of obtaining low and high yields and the minimum and maximum yield potential of tomato and maize in the maize-tomato rotation in three systems from 1994 to 2017.

Crop	System	Probability of low yield (<10 percentile)	Probability of high yield (>90 percentile)	Minimum yield potential (Mg ha^−1^)	Maximum yield potential (Mg ha^−1^)
Tomato	ORG	3.8% *	2.1%	67.201	75.179
	CONV	12.4%	16.6%	49.082	91.820
	CONV + WCC	19.2%	22.3% *	40.581	99.682
Maize	CONV	—	—	10.045	13.465
	CONV + WCC	—	—	6.483	8.616
	ORG	—	—	5.693	9.209

CONV: conventional maize-tomato system; CONV + WCC: maize-tomato system with winter cover crops; ORG: organic maize-tomato system. Stars represent significant differences from the random distribution based on left-tail tests (probability of low yield) or right-tail tests (probability of high yield) over 5000 iterations at the 0.05 significance level.

### Maximum yield potential

The potential of tomato and maize to obtain high yields was the highest in CONV + WCC for tomato and in CONV for maize. In the case of tomato, the probability of high yield (>90^th^ percentile) in CONV + WCC was significantly greater than the random distribution (pseudo-p = 0.038), and CONV + WCC showed the highest yield potential relative to the other two systems under the favorable condition (i.e., highest EI) (Table 2). For maize, the potential of CONV to achieve maximum yields under favorable growing conditions was higher than the other two systems (Table 2).

## Discussion

Although resilience has been proposed as an important feature for agricultural systems in the face of future climate change, it is often more discussed as a theory rather than a foundation for quantitative tools to monitor changes in system dynamics^35,36^. We provide here the first comparative analysis of yield resilience of an irrigated rotation system under conventional management and two intensities of ecological intensification management practices: organic management and the inclusion of cover crops. We proposed and implemented a novel yield resilience assessment framework, which quantifies four core aspects of long-term yield dynamics: absolute yields, yield stability, yield resistance, and maximum potential. This framework will allow better integration of resilience and stability metrics into agroecosystem performance assessments based on long-term yield dynamics^4^ and provide strong foundation for long-term comparisons of systems and management approaches.

**Figure 4:**
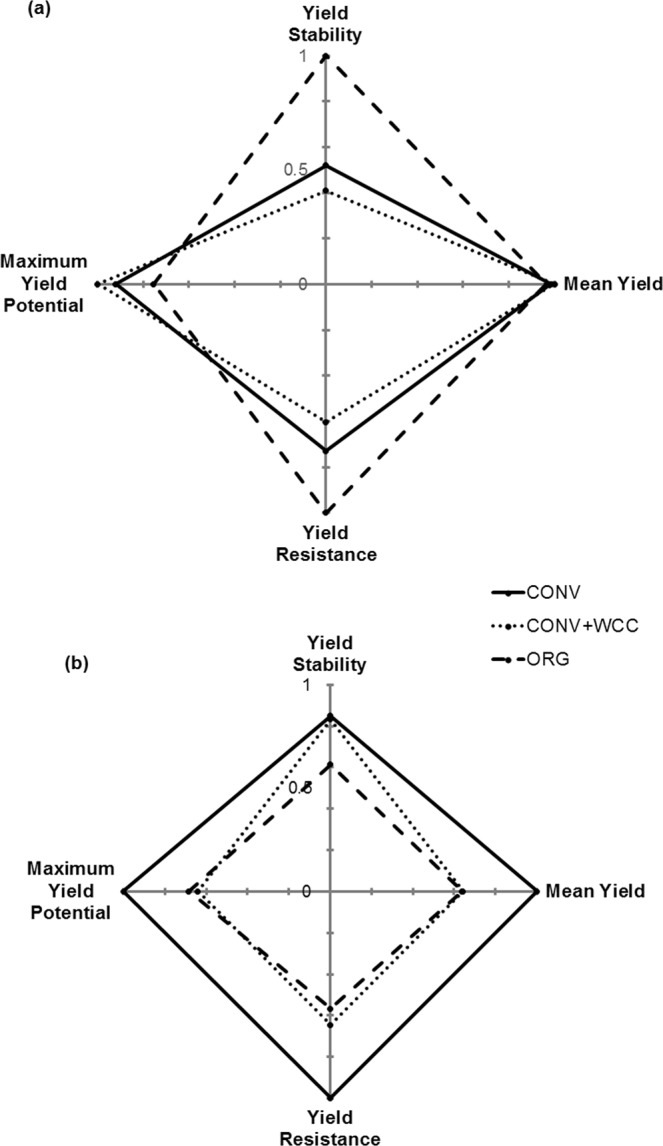
Summary of yield resilience of tomato (**a**) and maize (**b**) in the conventional maize-tomato system (CONV), maize-tomato system with winter cover crops system (CONV + WCC), and organic maize-tomato system (ORG). Solid lines = CONV; dotted lines = CONV + WCC; and long-dashed lines = ORG. Values represent the ratio of the performance of target system relative to the maximum across three systems. Yield stability is the average ratio of four stability metrics (see Table 1). Yield resistance is the ratio of minimum yield potential (see Table 2).

This framework was successful in identifying crop-specific resilience responses to three management systems. Our results show that benefits of organic management extended beyond average yields and provided important leverages to maintain high yield stability and mitigate the risk of low tomato yields during stressful growing conditions (Fig. 4a). However, these effects were crop dependent, and organic system was the least resilient among three management systems for maize in the same maize-tomato rotation (Fig. 4b). Despite the maize dataset not being as complete, the different long-term yield resilience patterns between a horticultural (tomato) and staple major cereal (maize) crop indicate that a larger array of crop response to management should be independently tested to design more resilient agricultural systems into the future. This information would fill the critical knowledge gap necessary to reorient policies and markets from favoring short-term productivity gains of a few major crops to reaching long-term nutritional, stability and resistance goals in more diversified systems.

Our hypothesis was that ecological intensification practices would allow comparable yields, improve yield stability, and reduce yield losses under adverse environmental conditions relative to conventional systems. The yield dynamics of tomato supported our hypothesis showing that ORG and CONV + WCC systems can achieve high yields and that ORG management is especially effective in boosting yield resilience in the form of stabilizing yields and reduced yield losses to unfavorable environmental conditions (Fig. 4a). High yield stability of tomato under organic management was consistent across all four yield stability metrics (Table 1) which encompassed multiple temporal aspects of stability^37^, from inter-annual variances to mean system responses to growth conditions. Of main importance to practitioner is our original risk analysis, which confirmed that organic tomato growers would substantially decrease the risk of yield losses and can obtain 36.0% higher yields than conventional systems in years of adverse environmental conditions (Table 2).

Improvements in soil abiotic and biotic properties and functioning in ecologically intensified systems may have contributed to improvements in yield stability of tomato and mitigation of yield losses under suboptimal environmental conditions. These effects were evident in years with adverse weather conditions (1995, 1999, and 2003), when excessive spring rainfalls and low temperature were observed (Supplementary Fig. S1). While conventionally managed systems showed substantial tomato yield reductions likely due to severe soil anaerobiosis and compaction^38^, excess spring rainfalls did not affect organic tomato yields. Improved soil water infiltration as a result of enhanced soil structure related to increased SOC^39,40^ may have facilitated the drainage of excess soil water and decreased the impacts of compaction.

Interestingly, yield dynamics of maize in three systems showed reverse trends with lower stability of organically grown maize yields and reduced mean yields and resistance in organic and conventional systems with cover crops. Different yield and variability responses of maize and tomato in organic and conventional cropping systems are in agreement with previous meta-analyses, in which yield and stability gaps in organic systems are a function of crop functional traits and management histories^15,41^. However, these results have to be put into context and carefully discussed since several management practices that differed among the three systems might have disproportionally affected maize. First, a short-season but pathogen-susceptible maize variety was used in ORG and CONV + WCC to allow for the inclusion of cover crop from 1994–2002. This short-season variety was likely lower-yielding than the long-season variety used in the CONV system, especially in light of both ORG and CONV + WCC being historically more prone to weed pressure and pathogen population build up (e.g. *Fusarium spp*.)^38^. Second, the annual N inputs in ORG (179 kg N ha^−1^) was lower than the CONV (235 kg N ha^−1^), and maize was not fertilized in the CONV + WCC from 1994 to 2007 (Supplementary Table S1). The reduced N inputs have likely limited the yields of maize in the ORG and CONV + WCC systems relative to the CONV system. Finally, the contrasting yield trends between maize and tomato could also stem from asynchrony of plant-specific nutrient uptake and soil nutrient availability. Maize takes up more than 50% of its crop N early during vegetative growth^42^. However, N uptake in processing tomatoes is usually slow in the early vegetative growth stages and accelerates during reproductive stages, with the highest rates of N uptake occurring about 60 to 70 days after transplanting^43^. This delayed nutrient uptake pattern in tomato may better match the slow-releasing pattern of organically derived soil N compare to maize^44,45^, which might have experienced insufficient N availability and uptake during peak N demand period. Collectively, these factors may have countervailed the benefits of improved soil properties and lead to decreased maize yield and yield resilience in ORG and CONV + WCC compared to the CONV systems.

Although system stability is of major importance, maintaining high yields and improving trends remain major targets. Organic systems tend to be lower-yielding than conventionally managed systems, and are often criticized for their inefficiency and inability to meet growing global food demands^46^. Meta-analyses of organic systems have reported yields on average 19–25% lower than conventional farming system^13–15^. In our study, we found no differences between system management in mean tomato yields across 24 years (Fig. 1), but rates of increase were slower under organic management compared to the other systems (Fig. 2). The relative contribution of technological developments to yield gains likely differ between systems. Our experiment spans two transitions between tomato varieties in 2005 and 2013 (Supplementary Table S1), both of which increased yields of all three systems. Varieties selected coincided with those grown widely in local fields, and the newer varieties reportedly contributed to the general tomato yield increase in California^47,48^. Conventional systems also received synthetic inputs that can effectively control acute yield reducing factors and likely contribute to greater yield trends over time. Finally, changes in irrigation and fertilizer application methods, especially after 2014, likely resulted in a slower rate of tomato yield increase in the organic system relative to the other two management approaches. Subsurface drip irrigation along with fertigation, which delivers water and fertilizers precisely and frequently to the root zone, favors conventional management and have been reported to improve tomato yields by an average of 17% compared to furrow irrigation in conventional systems^49,50^. Organic systems rely on microbially mineralized nutrients from organic sources which may not have been as effective as fertigation in delivering and supplying nutrients to crops^49^. Further technological innovations for organic systems, such as improved irrigation and fertilization methods, may therefore help reduce the yield gap observed. Notably, technology developments and management innovations may significantly influence yield responses of different management systems in a short-term, but their impacts on system resilience require longer term monitoring. For instance, it has been shown that subsurface drip irrigation can rapidly decrease essential soil services, such as soil carbon accumulation and aggregation^49^, which sustain yields in organic systems. Technological advances must, therefore, be carefully considered in these systems to avoid unexpected tradeoffs and sacrifice soil health-building processes for short-term yield benefits.

Interestingly, during periods when tomato varieties and fertilization rates went unchanged (i.e., 2005–2012), organic tomatoes showed a significantly positive yield trend while the CONV system yield trend remained constant (Supplementary Table S3). This positive yield trend is likely a result of evolving management experience^51^ and gradual improvements in soil properties, especially SOC. The substantial C additions in the form of composted manure and cover crops in the organic system has increased SOC by 32.6% in the first ten years of the experiment without showing signs of leveling off, whereas SOC increased only by 2.3% in the CONV system^52^. This increase in SOC was highly correlated with increases in soil health metrics such as soil aggregate stability, soil organic N, and microbial biomass^53,54^. The addition of composted manure could also support diverse microbial and invertebrate communities that in turn promote nutrient cycling^55^. These improvements in soil ecosystem services and functioning may have supported increased tomato yield trends in the time of no technological gains in organic systems.

Inclusion of cover crops before tomatoes showed comparable absolute yields and the highest potential of maximizing tomato yields under beneficial conditions, although it also showed the highest yield variability across the three systems. High yield variability in conventional systems with cover crops may be attributed to the combined effects of weather and the timing of cover crop termination, which strongly affect cover crop growth and decomposition rate and potential interference with cash crop planting and nutrient release^7^. Our results show that mixed approaches with the inclusion of cover crops and synthetic fertilizers may provide opportunities to better capitalize on favorable environmental conditions for crop production. However, thorough understandings of the timing and intensity of the management practices are required to maximize the beneficial effects of the mixed approaches to match short-term crop nutrient demands, while reducing long-term yield variability and mitigating nutrient loss and other environmental problems^23,44,56^.

In conclusion, the focus of agricultural studies needs to move away from yield-maximization centered approaches to equally stress system resilience in the future. Our proposed framework of integrating yields, yield temporal stability, yield resistance to unfavorable conditions, and yield potential to maximize production to optimal conditions, provide insights for comparing yield resilience of different management systems. We show the potential of long-term organic management systems to achieve high yields and resiliency to environmental stressors for tomatoes. Rewarding growers of horticultural crops through lower crop insurance premiums for risk mitigation strategies that both build soil health and resilience could incentivize the adoption of these practices. However, crop varieties and technological improvements tailored to system-specific ecosystem functioning are needed to enhance adoption and maximize the multiple benefits of more sustainable systems. In particular, cereals such as maize often exhibit substantial yield and stability reductions in organic systems compared to conventional systems, likely due to the extensive efforts to breed for high-yielding cereal varieties adapted to intensive synthetic inputs^15^. Varieties that are custom-designed for organic systems – i.e. those that can capitalize on root-microbe interactions, are adapted to slow-release nutrients, and are resistant to diseases and weeds – may contribute to close the yield gap^57^. These discrepancies also call for further research to determine crop-specific responses to long-term changes in soil health, and integrate agronomic efforts to further reduce maize yield gaps and prime organic maize systems for resilient responses to stressful environmental conditions^15,21,58^. Finally, future experimental studies with longer experimental duration along with modeling approaches will provide opportunities to optimize the resilience framework and help identify optimal management systems for different crops to increase resilience in the long run. Measuring long-term effects of different management practices on multidimensional ecosystem outcomes, including productivity, resilience, and ecosystem services will facilitate the design of integrated agricultural systems combining successful practices.

## Supplementary information

Supplementary information

## Data Availability

The datasets generated during and/or analyzed during the current study are available from the corresponding author on reasonable request.
