# Supplementary material for: Yields and resilience outcomes of organic, cover crop, and conventional practices in a Mediterranean climate
Source: Sci Rep. 2019 Aug 22;9:12283. doi: 10.1038/s41598-019-48747-4 (PMC6706438; doi:10.1038/s41598-019-48747-4)
Supplement: Supplementary file 1 — Supplementary information [file 41598_2019_48747_MOESM1_ESM.pdf]

Supplementary information for:

Yields and resilience outcomes of organic, cover crop, and conventional practices in a Mediterranean climate

Meng Li[1], Caitlin A. Peterson[1], Nicole E. Tautges[2], Kate M. Scow[3], and Amélie C. M. Gaudin[1, *]

[1] Department of Plant Sciences, University of California, Davis, One Shields Avenue, Davis, CA 95616, United States

[2] Agricultural Sustainability Institute, University of California, Davis, One Shields Avenue, Davis, CA 95616, United States

[3] Department of Land, Air, and Water Resources, University of California, Davis, One Shields Avenue, Davis, CA 95616, United States

* Corresponding author: agaudin@ucdavis.edu

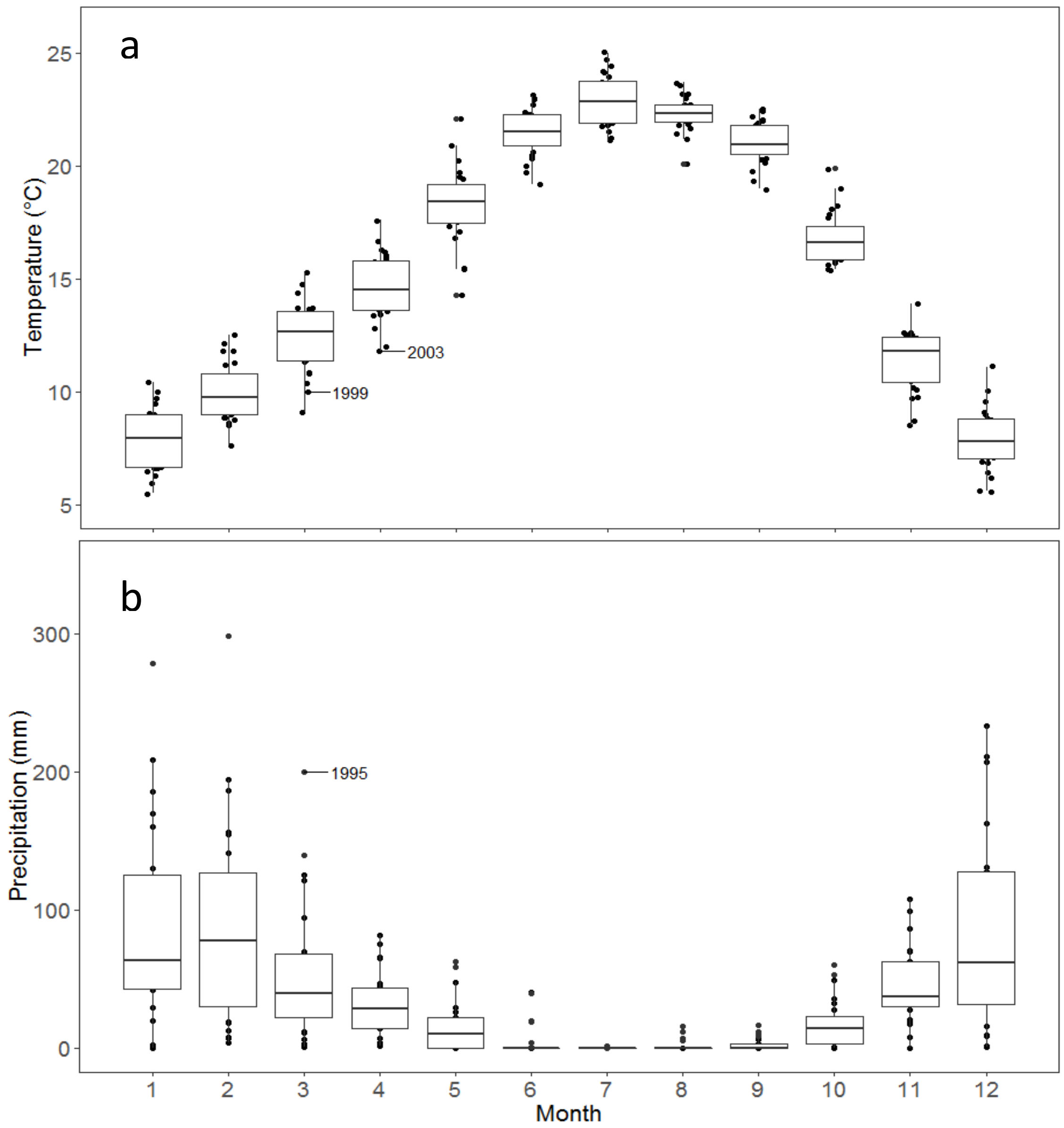

Supplementary Figure S1. Monthly average environmental conditions at the experimental site.

(a) Monthly air temperature and (b) precipitation are shown for the period of 1994-2017.

Supplementary Table S1. Information of crop variety, nitrogen (N) inputs, and winter cover crops in conventional maize-tomato (CONV), maize-tomato with winter cover crop (CONV+WCC), and organic maize-tomato system (ORG) from 1994 to 2017.

**Year**		**Tomato**<br>**Crop Variety**			**N input (kg ha^{-1})**		**Winter cover crop**	
	CONV	CONV+WCC	ORG	CONV	CONV+WCC	ORG	CONV+WCC	ORG
1994	Hailey 3155	Hailey 3155	Hailey 3155	135.7	135.7	179.3	NA	Legume
1995	Hailey 3155	Hailey 3155	Hailey 3155	237.3	237.3	201.8	NA	Legume
1996	Hailey 3155	Hailey 3155	Hailey 3155	159.3	159.3	134.5	NA	Legume
1997	Hailey 3155	Hailey 3155	Hailey 3155	161.7	161.7	179.3	NA	Legume
1998	Hailey 3155	Hailey 3155	Hailey 3155	159.4	159.4	NA	NA	Legume
1999	Hailey 3155	Hailey 3155	Hailey 3155	113.2	113.2	NA	NA	Legume
2000	Hailey 3155	Hailey 3155	Hailey 3155	158.4	158.4	179.3	NA	Legume
2001	Hailey 3155	Hailey 3155	Hailey 3155	158.4	158.4	179.3	NA	Legume
2002	Hailey 3155	Hailey 3155	Hailey 3155	162.6	162.6	179.3	NA	Legume
2003	Hailey 3155	Hailey 3155	Hailey 3155	168.2	168.2	179.3	NA	Legume
2004	Hailey 3155	Hailey 3155	Hailey 3155	162.6	50.4	179.3	Legume	Legume
2005	Heinz 9780	Heinz 9780	Heinz 9780	162.6	50.4	179.3	Legume	Legume
2006	Heinz 9780	Heinz 9780	Heinz 9780	162.6	50.4	179.3	Legume + Cereal	Legume + Cereal
2007	Heinz 9780	Heinz 9780	Heinz 9780	162.6	50.4	179.3	Legume + Cereal	Legume + Cereal
2008	Heinz 9780	NA	Heinz 9780	162.6	NA	179.3	Legume + Cereal	Legume + Cereal
2009	Heinz 9780	Heinz 9780	Heinz 9780	162.6	162.6	179.3	Legume + Cereal	Legume + Cereal
2010	Heinz 9780	Heinz 9780	Heinz 9780	162.5	162.5	179.3	Legume + Cereal	Legume + Cereal
2011	Heinz 9780	Heinz 9780	Heinz 9780	162.5	162.5	179.3	Legume + Cereal	Legume + Cereal
2012	Heinz 9780	Heinz 9780	Heinz 9780	162.5	162.5	179.3	Legume + Cereal	Legume + Cereal
2013	Heinz 8504, conv.	Heinz 8504, conv.	Heinz 8504, org.	134.5	134.5	179.3	Legume + Cereal	Legume + Cereal
2014	Heinz 8504, conv.	Heinz 8504, conv.	Heinz 8504, org.	200.0	200.0	179.3	Legume + Cereal	Legume + Cereal
2015	Heinz 8504, conv.	Heinz 8504, conv.	Heinz 8504, org.	200.0	200.0	179.3	Legume + Cereal	Legume + Cereal
2016	Heinz 8504, conv.	Heinz 8504, conv.	Heinz 8504, org.	200.0	200.0	179.3	Legume + Cereal	Legume + Cereal
2017	Heinz 8504, conv.	Heinz 8504, conv.	Heinz 8504, org.	200.0	200.0	179.3	Legume + Cereal	Legume + Cereal

Supplementary Table S1. Continued.

Year	Maize							
	**Crop Variety**			**N input (kg ha^{-1})**			**Winter cover crop**	
	CONV	CONV+WCC	ORG	CONV	CONV+WCC	ORG	CONV+WCC	ORG
1994	Pioneer 3162	NC +4616	NC +4616	314.4	0.0	358.7	Legume	Legume
1995	NC +4616	NC +4616	NC +4616	280.8	0.0	538.0	Legume	Legume
1996	Pioneer 3162	NC +4616	NC +4616	235.5	0.0	394.5	Legume	Legume
1997	Pioneer 3162	NC +4616	NC +4616	243.2	0.0	313.8	Legume	Legume
1998	Pioneer 3162	NC +4616	NC +4616	225.7	0.0	NA	Legume	Legume
1999	NC +4616	NC +4616	NC +4616	228.9	0.0	NA	Legume	Legume
2000	Pioneer 3162	NC +4616	NC +4616	243.7	0.0	179.3	Legume	Legume
2001	Pioneer 3162	NC +4616	NC +4616	225.7	0.0	179.3	Legume	Legume
2002	Pioneer 3162	NC +4616	NC +4616	218.5	0.0	179.3	Legume	Legume
2003	ST7570RR	ST7570RR	ST7570	235.5	0.0	179.3	Legume	Legume
2004	ST7570RR	ST7570RR	ST7570	235.5	0.0	179.3	Legume	Legume
2005	ST7570RR	ST7570RR	ST7570	235.5	0.0	179.3	Legume	Legume
2006	ST7570RR	ST7570RR	ST7570	235.5	0.0	179.3	Legume + Cereal	Legume + Cereal
2007	ST7570RR	ST7570RR	ST7570	235.5	0.0	179.3	Legume + Cereal	Legume + Cereal
2013	Nutech 0A-717	Nutech 0A-717	Blue River hybrids, 76H50	235.5	235.5	179.3	Legume + Cereal	Legume + Cereal
2014	Nutech 0A-717	Nutech 0A-717	Blue River hybrids, 76H50	235.5	235.5	179.3	Legume + Cereal	Legume + Cereal
2015	Nutech 0A-717	Nutech 0A-717	Blue River hybrids, 76H50	235.5	235.5	179.3	Legume + Cereal	Legume + Cereal
2016	Nutech 0A-717	Nutech 0A-717	Blue River hybrids, 76H50	235.5	235.5	179.3	Legume + Cereal	Legume + Cereal
2017	Nutech 0A-717	Nutech 0A-717	Blue River hybrids, 76H50	235.5	235.5	179.3	Legume + Cereal	Legume + Cereal

Supplementary Table S2. Analysis of variance for the yield of tomato and maize as predicted by system, year, and their interaction.

Crop	Year	Source	Degree of freedom	F-value	P-value
Tomato	1994-2017	System	2	0.616	0.541
		Year	1	244.902	<0.001
		System * Year	2	7.591	<0.001
Maize	1994-2007	System	2	167.553	<0.001
		Year	1	64.733	<0.001
		System * Year	2	16.553	<0.001
	2012-2017	System	2	22.830	<.001
		Year	1	0.019	0.891
		System * Year	2	1.414	0.256

Supplementary Table S3. Yield trends of tomato and maize in years with no variety change in conventional maize-tomato (CONV), maize-tomato with winter cover crop (CONV+WCC), and organic maize-tomato system (ORG) from 1994 to 2017.

Crop	Year	System	Yield trends (Mg ha^{-1} per year)	P-value
Tomato	1994-2004	ORG	1.150	0.060
		CONV+WCC	0.320	0.710
		CONV	1.620	0.114
	2005-2012	ORG	2.250	0.030
		CONV+WCC	8.840	<0.001
		CONV	0.270	0.660
	2013-2017	ORG	-1.611	0.515
		CONV+WCC	4.052	0.446
		CONV	2.767	0.286
Maize	1994-2002	ORG	-0.405	0.001
		CONV+WCC	-0.120	0.110
		CONV	0.090	0.470
	2003-2007	ORG	0.027	0.946
		CONV+WCC	-0.600	0.013
		CONV	-0.280	0.390
